# Post transcriptional control of the epigenetic stem cell regulator PLZF by sirtuin and HDAC deacetylases

**DOI:** 10.1186/s13072-015-0030-8

**Published:** 2015-09-24

**Authors:** Melanie J. McConnell, Laetitia Durand, Emma Langley, Lise Coste-Sarguet, Arthur Zelent, Christine Chomienne, Tony Kouzarides, Jonathan D. Licht, Fabien Guidez

**Affiliations:** Malaghan Institute for Medical Research, P.O. Box 7060, Wellington, New Zealand; INSERM UMRS-1131, Institut universitaire d’Hématologie, Université Paris Diderot, 1 avenue Claude Vellefaux, hôpital Saint-Louis, 75010 Paris, France; Wellcome Institute/Cancer Research UK, Department of Pathology, University of Cambridge, Tennis Court Road, Cambridge, CB2 1QR UK; Division of Hematology/Oncology, Feinberg School of Medicine, Northwestern University, Chicago, IL 60611 USA; Division of Hemato-oncology, Miller School of Medicine, Miami, FL 33136 USA; Division of Hematology/Oncology, Mount Sinai School of Medicine, New York, NY 10029 USA; Biogen Idec, San Diego, CA 92122 USA

**Keywords:** Repression, DNA methylation, Deacetylation, Epigenetic

## Abstract

**Background:**

The transcriptional repressor promyelocytic leukemia zinc finger protein (PLZF) is critical for the regulation of normal stem cells maintenance by establishing specific epigenetic landscape. We have previously shown that CBP/p300 acetyltransferase induces PLZF acetylation in order to increase its deoxynucleotidic acid (DNA) binding activity and to enhance its epigenetic function (repression of PLZF target genes). However, how PLZF is inactivated is not yet understood.

**Results:**

In this study, we demonstrate that PLZF is deacetylated by both histone deacetylase 3 and the NAD+ dependent deacetylase silent mating type information regulation 2 homolog 1 (SIRT1). Unlike other PLZF-interacting deacetylases, these two proteins interact with the zinc finger domain of PLZF, where the activating CBP/p300 acetylation site was previously described, inducing deacetylation of lysines 647/650/653. Overexpression of histone deacetylase 3 (HDAC3) and SIRT1 is associated with loss of PLZF DNA binding activity and decreases PLZF transcriptional repression. As a result, the chromatin status of the promoters of PLZF target genes, involved in oncogenesis, shift from a heterochromatin to an open euchromatin environment leading to gene expression even in the presence of PLZF.

**Conclusions:**

Consequently, SIRT1 and HDAC3 mediated-PLZF deacetylation provides for rapid control and fine-tuning of PLZF activity through post-transcriptional modification to regulate gene expression and cellular homeostasis.

## Background

Promyelocytic leukemia zinc finger protein (PLZF, ZBTB16), a transcription factor containing a bric à brac-tramtrack-broad complex (BTB) multimerization/repression domain and 9 zinc finger (ZF) motifs, belongs to a large family of transcriptional repressor proteins [1, 2]. First identified as a fusion partner of retinoic acid receptor alpha (RAR) in acute promyelocytic leukemia (APL) in cases with a *t*(11; 17) translocation [3], PLZF is now generally described as a transcriptional repressor and, more precisely, an epigenetic regulator. The biological activity of PLZF include its ability to regulate hematopoietic stem cell quiescence [4–6], maintain spermatogenesis [6–8] and induce the formation of specialized natural killer T cells (NKT) [9, 10]. Both direct and indirect PLZF functions have been elucidated and range from control of retrotransposon levels and mobility, to angiogenesis and cell signaling [6, 11, 12]. Loss of normal PLZF function in myeloid cells bearing the *t*(11; 17) is part of the oncogenic mechanism in acute promyelocytic leukemia development [13–15] while loss of expression of PLZF in melanoma [16], and a wide variety of solid tumors, including breast, prostate and glioblastoma (http://www.oncomine.org), suggest that PLZF may be a bona fide tumor suppressor.

Like a growing number of transcription factors such as p53 [17] and the related BTB/ZF factor Bcl-6 [18], the PLZF protein is regulated by acetylation of lysine residues. Unlike BCL6, where acetylation is associated with degradation of the Bcl-6 protein, and loss of BCL6 repression, acetylation of PLZF enhances DNA binding and subsequent repression of gene expression by PLZF [19]. We previously showed that CBP/p300 is the acetyltransferase protein that binds to PLZF, and acetylates several lysine residues in the zinc finger DNA binding domain. Loss of PLZF acetylation blocks PLZF repressor function and leads to global and specific hypomethylation of the mouse genome as well as impairment of hematopoietic and germinal stem cells maintenance [6]. While interaction with CBP/p300 triggers PLZF activation, the counterpart PLZF deacetylation and thus its inhibition mechanisms are still unknown.

A dynamic interplay between acetylation and deacetylation has been described [20]. The reciprocal deacetylation of proteins is carried out by the histone deacetylase enzyme family (HDAC) now referred to as protein deacetylases [21]. Not surprisingly, acetylation/deacetylation mechanisms are disrupted in cancer [22–24] and can modulate cell proliferation and cellular immunity [25]. The HDAC enzymes are divided into three classes. The class I consist of nuclear HDACs, which together with class II HDACs, shuttle between cytoplasm and nucleus. They share sequence similarity, are Zn^2+^ dependent deacetylases and regulate histone and non-histone protein functions [21]. The class III HDACs are sirtuins, NAD+ dependent deacetylases (SIRT 1–7), structurally unrelated to the classical HDAC I and II families [26–28]. Silent mating type information regulation 2 homolog 1 (SIRT1), a member of the sirtuin family, deacetylates non-histone proteins including transcription factors like p53, c-myc (reviewed in [29, 30]). Sirtuins have complex functions but are centrally involved in monitoring cellular metabolism and redox status, particularly in ageing [28, 31, 32]. SIRT1 is consistently over-expressed in acute myeloid leukemia [33, 34] while its expression is low in human and mouse bone marrow progenitor cells [35]. Interaction of PLZF with classes I and II HDACs, including HDAC1, 2, 4, 5, 6 and 9, to PLZF bound chromatin complex mediates PLZF’s epigenetic suppressor function [13, 36–39]. The BTB multimerization/repression domain of PLZF is in part accountable for the recruitment of co-repressor complexes including deacetylase proteins [13, 39], while the zinc finger motifs of PLZF tether these protein complexes to specific genetic targets. Curiously, HDAC3 is the only member of the class I family, that is neither recruited on DNA by nor associated with PLZF repression [6]. However, recent studies have shown that PLZF can directly interact with HDAC3 in order to form a corepressor complex interacting with NF-kB at the promoters of early inflammatory response genes [40, 41]. Furthermore, the nuclear-localized SIRT1 is not associated with PLZF repressor function but nevertheless binds to a fragment of the zinc finger domain of PLZF similar to that we had previously described for the RING finger protein promyelocytic leukemia protein (PML) ([42] and data not shown).

In this work we describe PLZF as a new substrate for HDAC3 and SIRT1 proteins. These interactions induce PLZF deacetylation resulting in subsequent loss of PLZF cellular localization, DNA binding and epigenetic function. These data identify the mechanisms controlling the acetylation/deacetylation cycle of the transcriptional activity of PLZF offering the possibility of fine tuning for its activity.

## Results

### SIRT1 and HDAC3 interact with PLZF zinc finger domain leading to its deacetylation

**Fig. 1:**
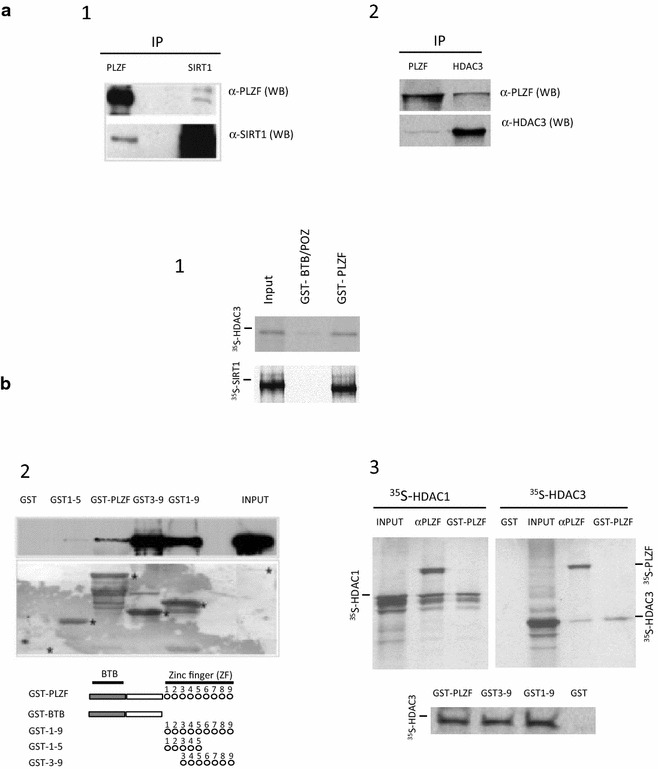
PLZF interacts with SIRT1 and HDAC3. Cellular coimmunoprecipitation of PLZF with deacetylases (**a**). Endogenous coimmunoprecipitation of PLZF and SIRT1 (**a.1**). Antibodies to PLZF and SIRT1 were used to precipitate each protein from 2 × 10^7^ KG1a cells as noted, and precipitates immunoblotted for PLZF (*top panel*) or SIRT1 (*bottom panel*) independently. Endogenous coimmunoprecipitations of PLZF and HDAC3 (**a.2**). Whole-cell extracts from KG1 cells were subjected to immunoprecipitation with anti-HDAC3 (HDAC3 IP) and anti-PLZF (PLZF IP) antibodies followed by immunoblotting with monoclonal antibodies raised against HDAC3 antibody (αHDAC3) and PLZF (αPLZF). In vitro mapping of PLZF interaction domains (**b**). Direct in vitro interaction between PLZF and SIRT1, and PLZF and HDAC3 was mapped by GST affinity chromatography using full length GST-PLZF. **b.1** Both SIRT1 and HDAC3 don’t interact with the N-terminus repressor domain of PLZF (GST-BTB/POZ). **b.2** *Top panel* bacterially expressed His-tagged SIRT1 was incubated with bacterially expressed GST, or GST-PLZF, subjected to electrophoretic separation and immunoblotted with an antibody against the HIS epitope tag. GST1–5, PLZF zinc fingers 1–5 only; GST-PLZF, full-length PLZF; GST3–9, zinc fingers 3–9 only; GST1–9, zinc fingers 1–9 only. Input, 5 % of volume of SIRT1 sample used in each pull-down. *Lower panel* ponceau staining of the blot, indicating amount of each protein loaded. *Asterisks* indicate the GST-fusion species in each lane. **b.3** Direct in vitro interaction was mapped by GST or in vitro immunoprecipitation using GST, GST-PLZF or ^35^S-labeled PLZF translated using the rabbit reticulocyte system (^35^S-PLZF) and incubated with ^35^S-labeled HDAC1 (^35^S-HDAC1) and HDAC3 (^35^S-HDAC3). *Bottom panel* GST pull-down using the GST3–9, zinc fingers 3–9; GST1–9, zinc fingers 1–9; GST-PLZF, full length PLZF and GST only. Anti-PLZF antibody (αPLZF) was used for coimmunoprecipitation to evaluate interactions between the PLZF, HDAC1 and HDAC3 proteins

**Fig. 2:**
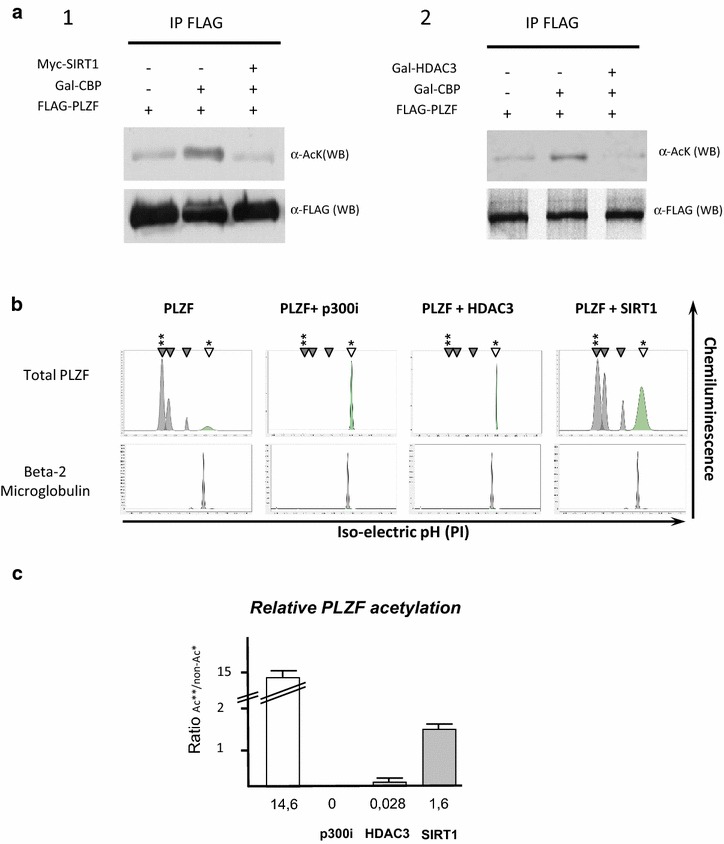
SIRT1 and HDAC3 deacetylase PLZF protein. Detection of PLZF by Western blotting (**a**). PLZF is deacetylated by SIRT1 (**a.1**). Expression constructs were transfected as indicated into 293T cells, whole cell lysates were extracted and immunoprecipitated with an antibody to the FLAG-tagged PLZF construct. Precipitates were immunoblotted with an antibody to acetylated PLZF species (*top panel* α-AcK) or FLAG (*bottom panel* α-FLAG). HDAC3 deacetylates PLZF (**a.2**). Expression constructs were transfected as indicated into 293T cells, whole cell lysates were extracted and immunoprecipitated with an antibody to the FLAG-tagged PLZF construct. Precipitates were immunoblotted with an antibody to acetylated PLZF species (*top panel* α-AcK) or FLAG (*bottom panel* α-FLAG). Detection of PLZF signatures by nanoimmunoassay. Electropherograms depicting levels of total and acetylated PLZF protein (**b**). Beta-2 microglobulin was used as loading control. Monoclonal PLZF antibody detects both acetylated (*grey arrows*) and non-acetylated (*green arrow*) forms as treatment by a p300 inhibitor (anacardic acid) induces the reduction of pics marked by *grey arrows.* Histogram plot (**c**) showing the ratio of acetylated versus non-acetylated forms of PLZF under the different conditions (PLZF only: PLZF; anacardic acid treatment: PLZF + p300i; co-expression of PLZF and HDAC3: PLZF + HDAC3 and co-expression of PLZF and SIRT1: PLZF + SIRT1). Each reaction was done in triplicate

It is well established that PLZF can interact with HDAC protein members through its BTB domain in order to mediated histone deacetylation at target genes. However, we have shown that HDAC3 and SIRT1 could also interact with PLZF ([13] and data not shown) suggesting that these specific interactions might serve a purpose other than to contribute to PLZF-mediated repression. An in vivo interaction between PLZF and SIRT1 (Fig. 1a.1) and between PLZF and HDAC3 (Fig. 1a.2) was observed by immunoprecipitation of endogenous proteins in human myeloid cells, KG1a. To characterize the domain within PLZF interacting with HDAC3 and SIRT1 in vitro interaction assays were performed. GST pull-down experiments confirmed that the full-length GST-PLZF protein could interact with His-SIRT1 and in vitro translated HDAC3 proteins (Fig. 1b). More detailed mapping of this interaction demonstrated that SIRT1 and HDAC3 do not interact with the BTB domain (Fig. 1b.1) but have a specific affinity for the C-terminal PLZF zinc fingers 3–9 (Fig. 1b.2, b.3, lower panel), the domain of PLZF previously shown to be acetylated by CBP/p300 on lysine residues located in zinc fingers 6 and 9 (ZF6 and ZF9) [19]. Indeed, CBP/p300-induced PLZF acetylation at its zinc finger 9 (ZF9) is a prerequisite for its repressor function [19] and its deacetylation is thus necessary to modulate its cellular activity [6]. To test the activity of these two deacetylase candidates, SIRT1 and HDAC3, 293T cells were transfected with FLAG-tagged PLZF, CBP/p300 acetyltransferase, HDAC3 and SIRT1 expression vectors. After PLZF immunoprecipitation and immunoblotting with an anti-acetyl-lysine antibody (α-AcK) to detect only the acetylated PLZF protein, we first confirmed that expression of CBP/p300 increases the amount of acetylated-PLZF as previously described [19] (Fig. 2a). Expression of either wild-type SIRT1 (Fig. 2a.1) or HDAC3 (Fig. 2a.2) reduced the quantity of acetylated PLZF, suggesting that PLZF could be a substrate of HDAC3 and SIRT1 deacetylases. In order to quantify the levels of PLZF acetylation in presence of these specific deacetylases, cell extracts were also subjected to a nanofluidic proteomic immunoassay in order to visualize the levels of PLZF acetylated isoforms. As shown in the electropherograms of Fig. 2b, the PLZF antibody detects both acetylated and non-acetylated PLZF isoforms. The presence of HDAC3, and in a lesser extent SIRT1, is associated with a decrease of detection of PLZF acetylation forms associated with an increased detection of the non-modified PLZF isoform (Fig. 2b, c).

### HDAC3 and SIRT1 antagonize PLZF DNA binding and transcriptional activities

**Fig. 3:**
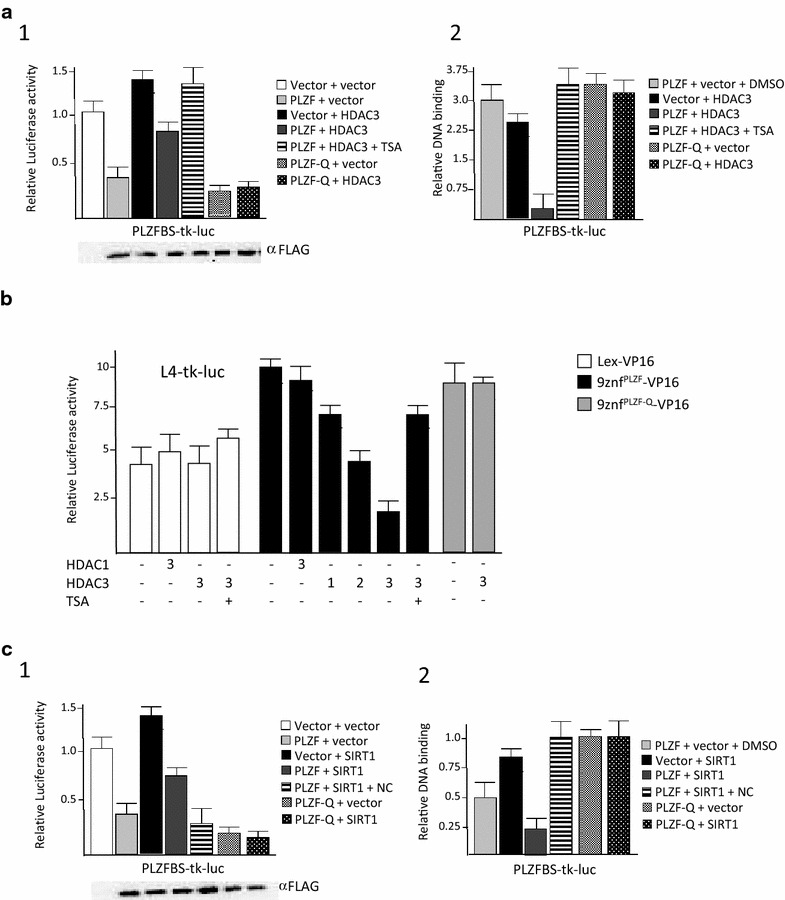
HDAC3/SIRT1-induced deacetylation affect PLZF DNA binding and transcriptional activities. HDAC3 over-expression influence PLZF activities in vivo (**a**). Transcriptional repression by PLZF is blocked by HDAC3 (**a.1**). 293T cells were transfected with construct as indicated. Cells were lysated at 18 h post transfection and luciferase assays performed. Luciferase activity is expressed relative to the activity in the vector only sample (*white bars*). PLZF alone, *light grey bars*. HDAC3 alone, *black bars*. PLZF and HDAC3, *dark grey bars*, PLZF and HDAC3 with TSA treatment, *striped bars* and PLZF-Q *mutant dotted bars*. Each experiment was performed in triplicate and the data represents the average of at least three experiments. *Errors bars* standard error of mean. *Lower panel* control of PLZF expression using immunoblotting detection with a FLAG antibody (*bottom panel* α-FLAG). Chromatin immunoprecipitation of the PLZF target (**a.2**). Flagged PLZF transfected 293T cells were treated with either DMSO or 20 nM Trichostatin A, and used for chromatin immunoprecipitation with an antibody against either FLAG or a IgG antibody control. For each condition, the amount of the HoxB2 promoter DNA spanning a PLZF binding site bound by each antibody was amplified and quantified by real-time PCR. This was expressed to the signal obtained from the 5 % input chromatin samples. Transcriptional PLZF binding activity is regulated by PLZF acetylation (**b**). The activity of the chimeric protein 9znf^PLZF^-VP16 was tested in transient transfection experiments and compare to the Lex-VP16 chimeric protein. Where indicated HDAC1 or HDAC3 expression vectors were cotransfected with increasing amount amount (1, 50 ng; 2, 100 ng, 3, 150 ng). Each experiment was performed in triplicate and the data represents the average of at least three experiments. *Errors bars* standard error of mean. SIRT1 over-expression influence PLZF activities in vivo (**c**). Transcriptional repression by PLZF is blocked by HDAC3 (**c.1**). 293T cells were transfected with constructs as indicated. Cells were lysed at 18 h post transfection and luciferase assays performed. Luciferase activity is expressed relative to the activity in the vector only sample (*white bars*). PLZF alone, *light grey bars*. SIRT1 alone, *black bars*. PLZF and SIRT1, *dark grey bars* and PLZF and SIRT1 with nicotinamide treatment, *striped bars*. Luciferase activity is expressed relative to the activity in the vector only sample (*white bars*). *Error bars* standard error of the mean. 2.5 kb of the c-myc promoter, −1.8 kb to +0.7 kb relative to the P1 promoter, 5′ to luciferase. *Lower panel* control of PLZF expression using immunoblotting detection with a FLAG antibody (*bottom panel* α-FLAG). Chromatin immunoprecipitation of the PLZF target (**c.2**). Transfected cells were treated with either DMSO or 10 mM nicotinamide, and used for chromatin immunoprecipitation with an antibody against PLZF For each condition, the amount of c-myc promoter DNA spanning a PLZF binding site bound by each antibody was amplified and quantified by real-time PCR. This was expressed relative to the signal obtained from the 5 % input chromatin sample

We previously showed that the PLZF protein has an increased affinity to DNA when acetylated and that mutation of PLZF lysines 647/650/653 to glutamine residues (PLZF-Q) mimics PLZF acetylation and binds constitutively to PLZF DNA targets [6, 19]. Therefore, we examined the ability of PLZF to bind to DNA in complementary in vitro assays in the presence of SIRT1 and HDAC3 deacetylases. Firstly, in a luciferase reporter assay the presence of HDAC3 partially blocked wild type PLZF-mediated repression (from 60 to 20 % repression activity) and not the PLZF-Q mutant (Fig. 3a.1), while expression of HDAC3 on its own had no effect on the reporter gene. Chromatin immunoprecipitation (ChIP) assays were employed to determine whether HDAC3 reduction of repression by PLZF was reflected by decreases in the occupancy of PLZF binding site. The result of this analysis shows a loss of wild type PLZF DNA binding activity, and not the PLZF-Q mutant, on the reporter plasmid when HDAC3 was co-expressed and, addition of HDAC3 inhibitor, Trichostatin A (TSA), restore PLZF ability to bound DNA (Fig. 3a.2). As shown above, treatment with the HDAC3 inhibitor TSA restored the capacity of PLZF to bind DNA but did not restore the ability of PLZF to repress the reporter gene (Fig. 3a.1, a.2 respectively). The latter finding may possibly be due to the concomitant inhibition by TSA of class I HDACs needed for PLZF-mediated repression. Finally, we used a chimeric PLZF, containing the nine zinc finger of PLZF fused to the VP16 activating domain, replacing the repression domain of the wild-type protein (9znf^PLZF^-VP16) [43]. This protein activates a PLZF binding site-containing reporter. Co-expression of increasing amounts of HDAC3 significantly decreases the activation of the reporter, likely due to the loss of binding of the 9znf^PLZF^-VP16 protein to DNA. This loss of activation was not noted in the presence of HDAC1 expression, or while using the zinc finger point mutants (PLZF-Q), and was corrected by TSA treatment in the presence of HDAC3 over-expression (Fig. 3b).

Similarly, co-expression of PLZF and SIRT1 relieved the PLZF-mediated repression, resulting in only a 25 % repression of luciferase activity compared to 70 % repression with PLZF in the absence of SIRT1 (Fig. 3c.1) associated with a decrease of PLZF binding site occupancy (Fig. 3c.2). Conversely, inhibition of SIRT1 with the sirtuin inhibitor, nicotinamide (NC) [44] significantly enhanced PLZF mediated repression (Fig. 3c.1) associated with a nine-fold enrichment of PLZF binding after SIRT1 inhibition (Fig. 3c.2).

### HDAC3/SIRT1-mediated deacetylation inhibits PLZF binding activity altering its cellular localization and blocking its epigenetic function

**Fig. 4:**
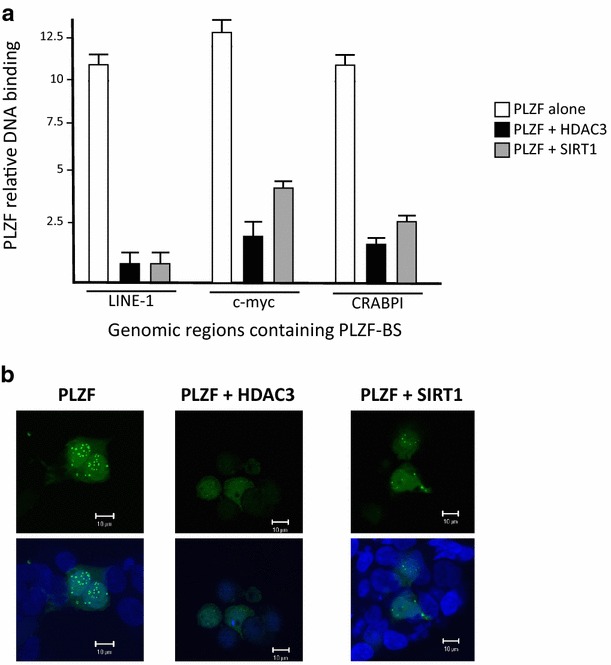
Effect of PLZF deacetylation. PLZF occupancy of its endogenous DNA binding sites (**a**). Transfected 293T cells were used for chromatin immunoprecipitation with an antibody against PLZF or an antibody against histone H3 as positive control. For each condition, the amount of LINE-1, CRABPI and c-myc DNA spanning a PLZF binding site bound by each antibody was amplified and quantified by real-time PCR. This was expressed relative to the signal obtained from the 5 % input chromatin sample. PLZF localizes in specific subnuclear compartments in the presence or absence of HDAC3 and SIRT1 deacetylases (**b**). The nuclear localization pattern of PLZF (and indicated conditions) was analyzed in 293T cells transfected with PLZF alone (PLZF) or in the presence of HDAC3 (PLZF + HDAC3) and SIRT1 (PLZF + SIRT1), by indirect immunofluorescence and confocal microscopy, as reported previously [19], punctate nuclear distribution of wild-type PLZF was observed (PLZF). Only diffuse nuclear localization was observed when PLZF was co-expressed with HDAC3 (PLZF + HDAC3), whereas co-expression of PLZF and SIRT1 (PLZF + SIRT1) show both diffuse and punctate localization (at a lesser degree than PLZF alone). No immunofluorescence signal was observed when primary anti-PLZF monoclonal antibody was omitted from the experimental procedure

The in vitro results of the reporter experiments containing the PLZF DNA binding sequences were corroborated by a ChIP assay on endogenous targets. We transfected PLZF into 293T cells and showed that PLZF could interact with previously identified genomic DNA targets (Fig. 4a, white bars). PLZF binding activity in vivo was decreased by co-transfection of HDAC3 and SIRT1 expression plasmid but not of an empty vector. The results of this analysis closely reflect those of the experiments assaying reporter gene activity (Fig. 3a.2, c.2) and those that indicate that PLZF is found acetylated when ectopically transfected in cells (Fig. 2). We then examined, whether the expression of HDAC3 and SIRT1 could impaired PLZF nuclear localization status, since we previously found that acetylation is necessary for PLZF to localize in a speckled nuclear pattern [19]. Immunofluorescence and confocal microscopy analyses indicated that coexpression of PLZF with HDAC3 or SIRT1 disrupts the punctate nuclear localization pattern of PLZF (Fig. 4b), resulting in a diffuse nuclear PLZF staining consistent with immunoblot results indicating no change in total PLZF protein (Fig. 2a.1, a.2). Taken together, these results suggest that PLZF cellular localization is directly linked to its physical interaction with DNA.

**Fig. 5:**
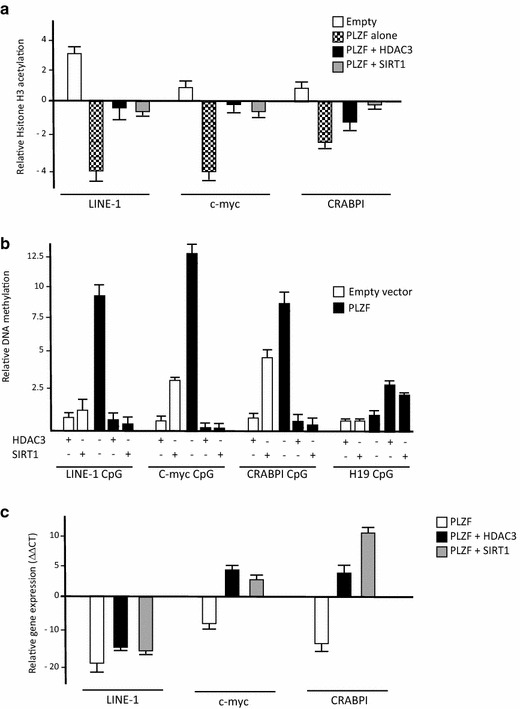
PLZF epigenetic effects on its endogenous targets and their related gene expression. Histone H3 enrichment at PLZF promoter targets (**a**). Transfected 293T cells were used for chromatin immunoprecipitation with an antibody against histone H3 or an antibody against acetylated forms of histone H3. For each condition, the amount of LINE-1, CRABPI and c-myc promoters bound by each antibody was amplified and quantified by real-time PCR. This was expressed relative to the signal obtained from the 5 % input chromatin sample and corrected by the signal obtained with the total histone H3 immunoprecipitation. DNA methylation enrichment of CpG promoters (**b**). MeDIP assay of the LINE-1, c-myc, CRABPI and H19 promoter regions in the PLZF with or without HDAC3 or SIRT1 expression cells. Cells were harvested 24 h after transfection and genomic DNA for MeDIP analysis with specific 5 mC antibody was isolated. Immunoprecipitated DNA was amplified by gene specific quantitative PCR. To quantify the amount of DNA methylation in these regions, the ratio of ΔCT of the MeDIP and input samples are calculated by comparing MeDIP samples against input (sonicated library DNA was set aside before MeDIP was performed for use as input DNA). The data are normalized to the DNA methylation at the UBE locus and fold enrichment ratio calculated in comparison to the untransfected cells. A representative dataset from these experiments, which were repeated 3 times, is shown. H19 locus was used as control of DNA methylation (known to be methylated in human) and as a non-PLZF targeted promoter. *White bars* represent transfection with the empty vector and the *black bar* with PLZF expression vector in presence (+) or absence (−) of HDAC3 or SIRT1 co-transfection. Relative gene expression of endogenous PLZF targets (**c**). To measure expression of PLZF targets, mRNA expression was measured at 12 h post-transfection in 293T cells using SYBR green quantitative real-time PCR. After normalization to GAPDH, expression levels of LINE-1, c-myc and CRABPI genes are presented as mean ± standard deviation. The experiments were conducted in triplicate

When bound to DNA, PLZF promotes heterochromatin formation by inducing epigenetic modifications including histone deacetylation and DNA methylation. Expression of the wild-type PLZF protein results in decrease of histone H3 acetylation in the region surrounding the PLZF binding sites of PLZF endogenous targets (Fig. 5a). However, expression of HDAC3 and SIRT1 alone do not alter significantly the level of histone H3 acetylation, their coexpression in presence of PLZF augment the levels of acetylated histone H3 indicating that deacetylation of PLZF by HDAC3 and SIRT1 directly affect PLZF-induced deacetylation at these sites (Fig. 5a). We have previously shown that PLZF recruitment can tether DNA methyltransferase activities (e.g. DNMT1) to specific DNA targets. Thus, PLZF induces DNA hypermethylation of CpG islands of the CRABPI gene and of the LINE-1 retrotransposon promoters [6, 15]. We performed methyl DNA immunoprecipitation (MeDIP) to assess the DNA methylation enrichment at PLZF target genes in absence or presence of HDAC3 and SIRT1. PLZF expression induced CpG hypermethylation of the c-myc, CRABPI and LINE-1 promoters, while HDAC3 and SIRT1 expression alone barely affected DNA methylation (Fig. 5b). However, co-expression of these deacetylases reduced significantly the hypermethylation induced by PLZF expression (Fig. 5b). Finally, to further confirm the inhibitory effects of HDAC3 and SIRT1 on PLZF activity, c-myc, CRABPI and LINE-1 expression was measured. Expression of either HDAC3, or SIRT1, was associated with an increase in expression of these PLZF target genes (Fig. 5c) and correlated with the change of epigenetic profiles at these promoters.

## Discussion

Post-translational modifications are crucial for regulating the functions of many eukaryotic proteins and among them, lysine acetylation has proven to be important for controlling transcription factor activity [45–47]. While acetylation of PLZF on lysine residues decisively leads to activation [6, 19], little is known about the inverse deacetylation mechanisms. In this study, we demonstrated a physical and functional interaction between PLZF and two subtypes of deacetylases, the histone Zn+ dependent deacetylase 3, HDAC3, and the NAD+ dependent deacetylase sirtuin, SIRT1. Unlike other reported deacetylases interacting with the repression domain of PLZF, these proteins interact with a specific domain (ZF 3–9) in the zinc finger region of PLZF corresponding to the interacting domain with CBP/p300 protein [19] including the activating acetylation motif (in ZF9). We have shown that these deacetylases effectively deacetylase PLZF in vitro and in vivo and that a specific mutant PLZF-Q, constitutively acetylated is not regulated by these deacetylases. PLZF-induced deacetylation leads to a decrease of its ability to interact interaction with endogenous DNA sequences, which in turn affects PLZF nuclear localization pattern. Interestingly, PLZF was recently reported to be acetylated at lysine 277 by the acetyltransferase HAT1 [41]. The acetylation at this site is necessary for PLZF to form a corepressor complex with HDAC3 and NK-kB in order to regulate inflammatory program [40, 41]. In this specific setting, PLZF doesn’t interact directly with DNA but is recruited to genomic targets through NF-kB interaction, indicating that specific PLZF acetylation could also affect PLZF function at different levels.

The cellular targets of PLZF play critical roles in cellular senescence c-myc [48], Retinoic acid-induced myeloid differentiation (CRABPI, [15]) and in the regulation of retrotransposon [6]. Over-expression of either HDAC3 and SIRT1 alter the PLZF-induced epigenetic profiles of CpG islands in these PLZF targets, leading to a decrease of DNA methylation and an increase in histone acetylation of these loci. Indeed, these promoters switch from heterochromatic feature (histone deacetylation/DNA methylation) to an euchromatic status (histone acetylation/DNA hypomethylation) in the presence of HDAC3 and SIRT1 deacetylases; ultimately, leading to de novo expression of PLZF target genes. Here, we have described the interplay between PLZF activity and HADC3/SIRT1 enzymatic functions, however, the characterization of this regulatory mechanism needs to be investigated in PLZF target tissues.

PLZF is expressed in hematopoietic stem cells (HSC) [4, 5] and generally represses hematopoietic development. Accordingly, we recently showed that gain or loss of PLZF acetylation is associated with a subsequent increase or loss of hematopoietic progenitors cells, respectively [6]. Loss of HDAC3 expression, which would lead to hyperacetylation and activation of PLZF, dramatically improves HSC (CD34+) cell expansion [49]. Likewise, SIRT1-deficient bone marrow cells confer stable bone marrow reconstitution in competitive repopulation and serial transplantation experiments [50]. Similarly, PLZF is a key factor involved in the maintenance of germinal stem cells [6, 8] and its expression is downregulated during spermatogenesis [51]. Recently, SIRT1 has also been described as a key factor involved in differentiation of male germ cells [52], indicating a possible interplay between PLZF and SIRT1 expression in order to regulate maintenance and differentiation of these cells.

To date, SIRT1 and HDAC3 appear to be complex regulatory factors with multiple roles in cell biology and transcriptional regulation and have been suggested as anti-cancers targets. HDAC3 is involved in the regulation of cancer-associated cellular process like apoptosis, and is also important in the regulation of cancer-associated transcription factors functions, including PCAF, SRY, NF-kB and STAT proteins [53]. SIRT1 deacetylates a growing list of non-histone proteins including transcriptional factors p53, NF-kB, nuclear receptors and c-myc [29, 30]. Here, we have shown that SIRT1 and HDAC3 bind to PLZF and negatively regulate its transcriptional activity suggesting a pivotal role in key cell function through PLZF [11]. Abnormal overexpression of these deacetylases could lead to the inhibition of PLZF repression and lead to proliferative advantage by up-regulation of c-myc, or increased genomic instability by reactivation of LINE-1 retrotransposons, both mechanisms shown to be involved in oncogenesis [54, 55]. This functional inhibition of PLZF is correlated with abnormal cytoplasmic localization and a recent study shows that high cytoplasmic detection of PLZF might be correlated with metastasis in thyroid carcinomas [56].

## Conclusions

Here we show that the acetylation site located in the zinc finger region of PLZF is a substrate of HDAC3 and SIRT1 deacetylases. When acetylated, PLZF binds to its DNA binding sites, and induces histone deacetylation and DNA hypermethylation followed by PLZF target genes repression. Specific deacetylation of PLZF by these deacetylases induces a loss of PLZF binding to its target genes, associated with epigenetic changes (e.g. histone acetylation and DNA hypomethylation) and ultimately leading to PLZF target genes expression. In conclusion, all the factors controlling both acetylation and deacetylation of PLZF are not well known, or are their effects well characterized, but their interplay will be critical for maintaining the balance of PLZF functions in cell differentiation and stem cell biology.

## Methods

### Protein affinity chromatography

Equivalent amounts of GST, GST-PLZF, or the various PLZF deletion constructs, each on beads, were incubated in 25 mM HEPES pH 7.5, 12.5 mM MgCl_2_, 150 mM NaCl, 20 % glycerol, 0.1 % NP40, 1 mM DTT, 20 μM ZnCl_2_, 3 µg BSA for 10 min at room temperature, before addition of recombinant His-SIRT1 for 1 h at room temperature. Beads were then washed 4× in 20 mM Tris pH 8.0, 100 mM NaCl, 1 mM EDTA, 0.5 % NP40. Samples were analyzed by SDS-PAGE followed by western blotting with anti-His antibody (Santa Cruz) and Ponceau staining of the membrane.

### In vitro immunoprecipitation

[35S]methionine-labeled proteins were synthesized in vitro using the TNT coupled transcription-translation system (Promega), following the supplier’s directions. Assays were performed in NETN buffer (20 mM Tris, pH 8.0, 100 mM NaCl, 1 mM EDTA, 0.5 % NP40) at 4 °C for 60 min with gentle rocking. Immunocomplexes were isolated by further incubation with an appropriate antibody preadsorbed on protein A/G-Sepharose (Pharmacia), washed five times in H buffer (20 mM HEPES, pH 7.7, 50 mM KCl, 20 % glycerol, 0.1 % NP40). Bound proteins were eluted in Laemmli loading buffer and separated on a 5 or 10 % SDS-PAGE. Gels were fixed in 25 % isopropanol and 10 % acetic acid, dried, and exposed to Kodak Biomax film. Anti-p300 (Santa Cruz Biotechnology), anti-Gal4 (Santa Cruz Biotechnology), rabbit polyclonal anti-acetyl-lysine (Upstate Biotechnology, catalog no. 06-933), and anti-Flag (Sigma) antibodies were purchased from the indicated suppliers and used as directed.

### Cell culture and transfection

KG1a cells were maintained in IMDM supplemented with 10 % heat-inactivated fetal bovine serum. For transfection of KG1a cells, 2 × 10^7^ cells per transfection were washed once in IMDM with no additives, resuspended in 400 μL of additive-free media and mixed with 20 μg of DNA in a 4 mm gap cuvette. Electroporation was carried out at 72 W, 220 V and 2800 μF in a BTX 600 electroporator (Genetronics, San Diego, CA, USA), the cells were allowed to recover at room temperature for 10 min, then plated into maintenance media. 293T cells were maintained in DMEM with 10 % heat-inactivated fetal bovine serum. 293T cells were plated 16 h before transfection with Superfect (Qiagen, Valencia, CA, USA). For every 1 microgram of plasmid DNA, 5 μL of Superfect was mixed with 90 μL of additive-free DMEM (Invitrogen, Carlsbad, CA, USA) and incubated at room temperature for 15 min. One mL of maintenance media was added and the DNA/Superfect/media mix was overlaid onto freshly washed 293T cells. This mix was removed after 3 h and replaced with maintenance media.

### Immunofluorescence and confocal microscopy

For immunofluorescence, cells were fixed in 4 % paraformaldehyde for 20 min at room temperature. Slides were then washed twice for 5 min in Ca^2**+**^- and Mg^2**+**^-free phosphate buffered saline (PBS) solution and cytospin onto polylysine-coated slides and permeabilized with 0.3 % Triton in PBS for 5 min at room temperature, washed twice for 5 min in PBS, and incubated in blocking buffer (1 % bovine serum albumin in PBS) for 30 min at room temperature. Cells were then incubated with mouse monoclonal anti-PLZF antibody (diluted 1:500 in blocking buffer) for 2 h at room temperature, followed by three 5-min washes in PBS. Secondary fluorescein isothiocyanate (FITC)-conjugated anti-mouse antibody (diluted 1:200 in blocking buffer) (Jackson ImmunoResearch Laboratories) was then applied for 2 h. Cells were subsequently washed twice for 5 min in PBS and then twice for 5 min in PBS plus To-pro3 iodide (dilution, 1:10,000). Cells were mounted in Vectashield mounting medium, sealed with nail varnish, and visualized using the Leica TCS SP2 true confocal system.

### Reporter assays

The luciferase reporter, PLZF and Sirt1 or HDAC3 expression plasmids were used in a 5:4:3 ratio respectively, with 10 ng of renilla luciferase included as an internal control for every microgram of plasmid DNA. 293T cells were transfected as described above. Transfected cells were harvested at 42–45 h post-transfection and lysates assayed for luciferase activity using the Dual Luciferase kit (Promega, Madison, WI, USA) as recommended by the manufacturer. Raw values obtained for each experimental point, performed in triplicate, were normalized to the renilla value for each replicate. The error for each experiment is represented by standard error of the mean for each triplicate. Data presented is from a representative experiment, the same effect was observed in at least three independent experiments.

### Quantitative RT-PCR

RNA was extracted from 0.5 × 10^5^ to 1 × 10^7^ cells using the RNeasy protocol, as recommended by the manufacturer (RNeasy, Qiagen, Valencia CA, USA), and cDNA produced using a mixture of random hexamers and oligo dT priming (iScript reverse transcriptase, Biorad, Hercules, CA, USA). Quantitative PCR was carried out in triplicate using the Quantitect SYBR Green master mix kit (Qiagen, Valencia, CA, USA) according to the manufacturers recommendations, in an Opticon DNA Engine (MJ Research, Reno, NV, USA). Primer sequences used are as follows:

- c-myc F: 5′-TCGGATTCTCTGCTCTCCTCG-3′
- c-myc R: 5′-CTGCGTAGTTGTGCTGATGTGTG-3′
- p21 F: 5′-ACAGCAGAGGAAGACCATGTGG-3′
- Line-1 F: 5′-GCTGGATATGAAATTCTGGGTTGA-3′
- Line-1 R: 5′-AGGAAATACAGAGAACGCCACAA-3′
- CrabpI F: 5′-GGACGCAAGTGCAGGAGTTTA-3′
- CrabpI R: 5′-GCGCCAAACGTCAGGATAA-3′
- GAPDH F: 5′-CCAAAATCAAGTGGGGCGATG-3′
- GAPDH R: 5′-AAAGGTGGAGGAGTGGGTGTCG-3′.

The cycle threshold (Ct) value for the ‘DMSO’ sample was taken as baseline expression, and ΔCt, the difference between the DMSO Ct and the Ct obtained after treatment, was calculated for each PCR. The ΔCt for each transcript was expressed relative to the ΔCt for GAPDH in each experiment. The formula 2ΔCt was used to calculate the fold change in gene expression after nicotinamide treatment. Efficiency of amplification was shown to be equivalent for all primers.

### Immunoprecipitation

For endogenous co-immunoprecipitation, whole cell lysates were prepared from 2 × 108 KG1a cells using 1 % NP40, 150 mM NaCl, 20 mM Tris pH 8.0 with a protease inhibitor cocktail (Complete, Roche, Indianapolis, IN, USA). Ten percent of each sample was reserved, and the remainder of the lysate was precleared by incubation with protein A agarose for 1 h at 4 °C. After centrifugation, the cleared lysate was divided up and incubated with 2 µg of either PLZF monoclonal antibody (EMD Biosciences, San Diego, CA, USA), HDAC3 monoclonal antibody (Santa Cruz Biotechnology, Santa Cruz, CA, USA) or anti-SIRT1 polyclonal antibody (Santa Cruz Biotechnology, Santa Cruz, CA, USA) as necessary for 16 h at 4 °C. Immune complexes were collected by incubation with 20 μL of protein A agarose for 1 h, washed three times in whole cell lysate buffer, and denatured by boiling in 40 μL of SDS loading buffer. For immunoprecipitation from nuclear extracts, the soluble nuclear fraction was obtained as previously described [19]. For anti-acetyl-lysine immunoprecipitations, each 10 cm plate of 293T cells was resuspended in 50 μL ice-cold PBS, 50 μL of 2 % SDS/PBS was added, and the lysates incubated at 95 °C for 10 min. The volume was taken to 1 mL with 1 % NP40, 150 mM NaCl, 20 mM Tris pH8 with a protease inhibitor cocktail (Complete, Roche, Indianapolis, IA, USA) and incubated 30 min on ice. Insoluble material was pelleted by centrifugation at 13,000 rpm for 15 min, and the supernatant retained for immunoprecipitation, which was carried out as described above with 1 μg of anti-acetyl lysine antibody (Merck Millipore) per sample.

### Chromatin and methyl DNA immunoprecipitation

Antibodies used were against PLZF (EMD Biosciences, San Diego, CA, USA), FLAG M2 (Sigma, St Louis, MO, USA), histone H3, pan-acetyl histone H3 and 5-methyl-cytosine (Abcam). For each immunoprecipitation, 293T cells were seeded at 1 × 10^5^/mL transfected and treated with either 10 mM nicotinamide, 10 mM of Trichostatin A or the appropriate volume of DMSO vehicle control for 18 h. Cells were then fixed in 1 % formaldehyde at room temperature for 30 min with shaking and quenched in 0.125 M glycine for 5 min at room temperature. Cells were washed twice in PBS containing complete protease inhibitor (Roche, Indianapolis, IN, USA) and lysed in 1.0 mL lysis buffer (140 mM NaCl, 10 mM Tris pH8, 1 % NP40) per 1 × 10^7^ cells. Lysates were sonicated to break DNA into fragments less than 1 kb (Dismembrator, Diagenode, Philadelphia, PA, USA), and pre-cleared for 45–60 min with protein A agarose beads with 0.4 μg/μL salmon sperm DNA (Upstate, Charlottesville, VA, USA). After brief centrifugation, supernatant was removed and incubated with 5 μg of the precipitating antibody overnight at 4 °C. Protein A/salmon sperm DNA was added, and the immune complex collected for 1 h at 4 °C. Complexes were washed for 5 min each in low salt buffer (0.1 % SDS, 1 % Triton-x100, 2 mM EDTA, 20 mM Tris–HCl pH 8.1, 150 mM NaCl), high salt buffer (0.1 % SDS, 1 % Triton-x100, 2 mM EDTA, 20 mM Tris–HCl pH 8.1, 500 mM NaCl), LiCl buffer (0.25 M LiCl, 1 % NP40, 1 % sodium deoxycholate, 1 mM EDTA, 10 mM Tris–HCl, pH 8.1), then washed twice in TE. DNA was eluted for 2 × 15 min in 250 μL 1 % SDS, 0.1 M NaHCO_3_, and the two eluates combined. 20 μL of 5 M NaCl was added and eluates incubated at 65 °C overnight to reverse the cross-links. DNA was recovered by phenol–chloroform extraction and ethanol precipitation, then used in a PCR reaction. The primer sequences used for PCR were:

- UBE2B promoter F: 5′-CTCAGGGGTGGATTGTTGAC-3′
- UBE2B promoter R: 5′-TGTGGATTCAAAGACCACGA-3′
- H19 ICR F: 5′-GAGCCGCACCAGATCTTCAG-3′
- H19 ICR R: 5′-TTGGTGGAACACACTGTGATCA-3′
- c-myc promoter F: 5′-AATGCCTTTGGGTGAGGGAC-3′
- c-myc promoter R: 5′-TCCGTGCCTTTTTTTGGGG-3′
- Line-1 PLZF-BS F: 5′-GAACTCTCCACCCCAAATCA-3′
- Line-1 PLZF-BS R: 5′-CCATGTAGTTGAGCGGCTTT-3′
- CrabpI PLZF-BS F: 5′-AGTCTCTATATAACAAGAGGCA-3′
- CrabpI PLZF-BS R: 5′-TCAGAACCATGTTAATTTTCCA-3′
- Line-1 CpG F: 5′-CGAATATTGCGCTTTTCAGA-3′
- Line-1 CpG R: 5′-CCGGCTGCTTTGTTTACCTA-3′
- CrabpI CpG F: 5′-ATTTCGACGAGCTGCTCAAG-3′
- CrabpI CpG R: 5′-CTACCAGCTTCTCCGAGACC-3′
- Luciferase reporter F: 5′-GGATCCCCACTTAACACCCAA-3′
- Luciferase reporter R: 5′-CTTGGGAAACTGCTCTTAACTAG-3′ (described in [15, 19]).

### Immunoblot analysis

Proteins were separated by 10 % SDS–polyacrylamide gel electrophoresis, and transferred to PVDF membrane (Millipore, Bedford, MA, USA) in a 25 mM Tris, 192 mM glycine buffer. The membrane was blocked in PBS/5 % skim milk powder overnight. Incubation of the membrane with the primary antibody was carried out at room temperature for 1 h in PBS/0.5 % skim milk, membranes were washed three times for 5 min in PBS, and the appropriate HRP-conjugated secondary antibody added to PBS at the concentration recommended by the manufacturer (Chemicon, Temecula, CA, USA). The HRP conjugate was detected by chemiluminescence using the ECL kit (Amersham, Piscataway, NJ, USA) and autofluorography. Antibodies used were against PLZF (EMD Biosciences, San Diego, CA, USA), SIRT1 (Santa Cruz Biotechnology), HDAC3 (Santa Cruz Biotechnology), the myc epitope tag (Santa Cruz Biotechnology), and GAPDH (Chemicon).

### Nanofluidic detection

The NanoPro 1000 system (ProteinSimple) is built on an automated, capillary-based immunoassay platform and enables a rapid and quantitative analysis of specific proteins and their post-translational modification states. We have utilized this nano-immunoassay to examine the acetylation profiles of the PLZF protein. All isoelectric separations were performed on the NanoPro 1000 (ProteinSimple, Santa Clara, CA, USA) with the Premix Generation 2 pH 3–10 separation mix (Cat #040–968). Standard p*I* Ladder 1 (ProteinSimple Cat #040–644) was added to the ampholyte pre-mix. Lysates were then separated for 40 min at 21,000 μW in individual capillaries. After separation the proteins in the lysate were immobilized to the capillary wall by subjecting them to UV exposure for a period of 80 s. After two washes of 150 s each, primary antibodies were introduced into the capillaries for a period of 2 h. Antibody for PLZF were used at a 1:75 dilution, whereas antibody for β-2 microglobulin were used at 1:100 dilutions. After another two washes of 150 s each, samples were run either with or without amplification reagents. Secondary anti-rabbit-HRP-conjugated antibodies (ProteinSimple Cat #040–656) or secondary anti-mouse-biotin-conjugated antibodies (ProteinSimple’s amplified mouse secondary antibody kit—Cat #041–127) were loaded into the capillary for 1 h. Amplification was performed only for PLZF antibody. After a third set of two washes of 150 s each, either streptavidin, conjugated with horse radish peroxidase (ProteinSimple Cat #041–126), or antibody diluent was loaded into the capillary for 2 h or 10 min respectively. After a final two wash cycle of 150 s each, a luminol-peroxidase 1:1 mix (ProteinSimple Cat #040–0652 and 040–684) was flowed through the capillaries and chemiluminescence was detected at 30, 60, 120, 240, 480, and 960 s. Primary monoclonal PLZF antibody (Abcam Ab104854) was used for the assay. To determine acetylation-peaks, sample lysates were treated with anacardic acid (AA) for 12 h (20 mM, Sigma SMB00129).

## Abbreviations

AcK: acetyl lysine
APL: acute promyelocytic leukemia
BTB: bric à brac-tramtrack-broad complex
Bcl-6: B-cell lymphoma 6 protein
CBP/p300: CREB binding protein/p300 protein
CD34: cluster of differentiation 34
ChIP: chromatin immunoprecipitation
c-myc: myc transcription factor
CpG: cytosine-phosphate-guanidine
CRABP: cellular retinoic acid binding protein
DNA: deoxynucleotidic acid
DNMT: DNA methyltransferase
HAT: histone acetyltransferase
HDAC3: histone deacetylase 3
HSC: hematopoietic stem cell
MeDIP: methyl DNA immunoprecipitation
NAD: nicotinamide adenine dinucleotide
NC: nicotinamide
NF-kB: nuclear factor kappa B
NKT: natural killer T
PCAF: p300/CBP assocoated factor
LINE-1: long interspersed element
p53: tumor protein p53
PLZF: promyelocytic zinc finger protein
PLZF-Q: PLZF protein containing mutation of lysines 647/650/653 to glutamine residues
PML: promyelocytic leukemia protein
RARA: retinoic acid receptor alpha
RING: really interesting new gene
SIRT: sirtuin (silent mating type information regulation 2 homolog) protein
SRY: sex-determining region Y
STAT: signal transducer and activator factor
TSA: tricostatin A
VP16: herpes simplex virus protein vmw65
ZF: zinc finger
Zn^2+^: zinc ion
